# Supplementary material for: Integrating Activity-Guided Strategy and Fingerprint Analysis to Target Potent Cytotoxic Brefeldin A from a Fungal Library of the Medicinal Mangrove Acanthus ilicifolius
Source: Mar Drugs. 2022 Jun 29;20(7):432. doi: 10.3390/md20070432 (PMC9315649; doi:10.3390/md20070432)
Supplement: Supplementary file 1 [file marinedrugs-20-00432-s001.zip › marinedrugs-1790937-supplementary.pdf]

Integrating Activity-guided Strategy and Fingerprint Analysis to Target Potent Cytotoxic Brefeldin A from a Fungal Library of the Medicinal Mangrove *Acanthus ilicifolius*

Cui-Fang Wang [1,2], Jie Ma [1,2], Qian-Qian Jing [1], Xi-Zhen Cao [1], Lu Chen [1], Rong Chao [1], Ji-Yong Zheng [2], Chang-Lun Shao [1,2,*], Xiao-Xi He[1,*], and Mei-Yan Wei [1,*]

[1] Key Laboratory of Marine Drugs, The Ministry of Education of China, School of Medicine and Pharmacy, College of Food Science and Engineering, Ocean University of China, Qingdao 266003, People's Republic of China; wangcuifang0115@163.com (C.-F.W.); jingqianqian1231@163.com (Q.-Q.J.); caoxizhen2022@163.com (X.-Z.C.); cl2014049048@163.com (L.C.); chaorong1122@163.com (R.C.); shaochanglun@163.com (C.-L.S.); mywei95@126.com (M.-Y.W.)

[2] State Key Laboratory for Marine Corrosion and Protection, Luoyang Ship Material Research Institute (LSMRI), Qingdao 266061, People's Republic of China; majie346@163.com (J. M.); zhengjy@sunrui.net (J.-Y.Z.);

[*] Correspondence: shaochanglun@163.com (C.-L.S.); hexiaoxi@ouc.edu.cn (X.-X.H.); mywei95@126.com (M.-Y.W.); Tel: +86-532-8203-1381 (M.-Y.W.);

Content of Supporting Information

Table S1. Phylogenetic affiliations of cultivable fungi associated with the *Acanthus ilicifolius*.

Figure S1. Neighbor-joining phylogenetic tree based on ITS sequences of cultured fungi associated with *Acanthus ilicifolius*.

Figure S2. Phylogenetic tree of fungi in the order *Hypocreales* based on ITS-rRNA sequence homology.

Figure S3. The cytotoxicity relative inhibition rate (%) data of the organic extracts in cancer cell lines.

Figure S4. The fingerprint analysis of the organic extract of fungi from different species and genera.

Figure S5. The fingerprint analysis of the organic extract of *Penicillium* sp. (HS-N-27).

Table S1. Phylogenetic affiliations of cultivable fungi associated with the *Acanthus ilicifolius*

Isolate ID	Order	Genus	Closest identified relative	Identity (%)	Querty cover (%)
HS-N-01	Hypocreales	*Trichoderma*	*Trichoderma* sp. (KM458790)	99	99
HS-Y-17			*Trichoderma reesei* (KU377472)	100	100
HS-N-04			*Trichoderma harzianum* (KT192387)	100	99
HS-N-26			*Trichoderma* sp. (KM066570)	100	100
HS-N-12		*Hypocrea*	*Hypocrea lixii* (HQ229942)	100	99
HS-N-02			*Hypocrea jecorina* (GU048859)	99	99
HS-J-15		*Acremonium*	*Acremonium* sp. (JQ717349)	99	97
HS-J-08			*Acremonium* sp. (HG936339)	99	98
HS-N-28		*Verticillium*	*Verticillium dahlia* (HQ839784)	99	99
HS-N-16			*Verticillium dahlia* (HQ839784)	99	99
HS-Y-08			*Verticillium dahlia* (HQ839784)	99	100
HS-N-08		*Fusarium*	*Fusarium solani* (KT211526)	100	100
HS-N-09			*Fusarium solani* (EU719658)	99	99
HS-N-10			*Fusarium solani* (EU719658)	99	99
HS-G-23			*Fusarium solani* (AY633744)	99	100
HS-G-15			*Fusarium solani* (AY633744)	99	99
HS-G-31			*Fusarium solani* (KT211549)	100	100
HS-Y-09			*Fusarium solani* (EU719658)	99	100
HS-J-06			*Fusarium incarnatum* (KU377454)	99	100
HS-J-19			*Fusarium* sp. (GQ410774)	99	100
HS-Y-20			*Fusarium* sp. (GQ352485)	99	99
HS-J-27			*Fusarium* sp. (GQ410774)	99	99
HS-J-09			*Fusarium proliferatum* (HQ380763)	99	100
HS-J-10			*Fusarium incarnatum* (KM921663)	99	99
HS-Y-06			*Fusarium* sp. (GQ352485)	99	100
HS-J-11			*Fusarium equiseti* (LT617634)	100	100
HS-N-31		*Neocosmospora*	*Neocosmospora vasinfecta*(JX997932)	99	99
HS-N-03	Eurotiales	*Penicillium*	*Penicillium janthinellum* (KU529846)	100	100
HS-N-22			*Penicillium janthinellum* (KM461118)	99	98
HS-N-23			*Penicillium janthinellum* (KM461118)	100	99
HS-G-06			*Penicillium chermesinum* (KJ767051)	95	100
HS-G-12			*Penicillium* sp. (KJ935029)	99	100
HS-N-27			*Penicillium* sp. (KF367521)	99	100
HS-N-29			*Penicillium* sp. (KU556495)	99	99
HS-G-24			*Penicillium* sp. (LT558883)	100	99
HS-G-30			*Penicillium oxalicum* (KM458784)	99	99
HS-N-15			*Penicillium chloroleucon* (KP016813)	99	98

HS-J-22			*Penicillium chrysogenum* (KX349473)	100	99
HS-J-26			*Penicillium brocae* (KJ775608)	100	97
HS-Y-01			*Penicillium* sp. (LT603036)	100	100
HS-Y-10			*Penicillium* sp. (FJ647577)	99	100
HS-Y-11			*Penicillium rubens* (KX011023)	98	99
HS-Y-04			*Penicillium sclerotiorum* (KC478540)	99	100
HS-G-01			*Penicillium* sp. (KR093905)	99	97
HS-N-19			*Penicillium* sp. (KT763472)	100	100
HS-G-20			*Penicillium* sp. (KT763472)	100	100
HS-N-20		*Eupenicillium*	*Eupenicillium* sp. (KC871024)	99	98
HS-N-25			*Eupenicillium* sp. (KC871024)	99	99
HS-G-29			*Eupenicillium* sp. (JQ828843)	99	99
HS-N-06		*Aspergillus*	*Aspergillus flavus* (KU597196)	99	99
HS-G-05			*Aspergillus terreus* (KC462061)	100	100
HS-G-07			*Aspergillus* sp. (JQ717354)	99	100
HS-G-16			*Aspergillus* sp. (KJ567459)	99	100
HS-Y-12			*Aspergillus* sp. (KX228408)	100	100
HS-N-30			*Aspergillus* sp. (JN393254)	100	99
HS-G-04			*Aspergillus versicolor* (LC105691)	99	99
HS-G-17			*Aspergillus* sp. (KF888648)	99	99
HS-Y-27			*Aspergillus aculeatus* (KU310905)	100	100
HS-G-03	Eurotiales	*Talaromyces*	*Talaromyces flavus* (KM458831)	99	97
HS-N-13	Xylariales	*Pestalotiopsis*	*Pestalotiopsis oxyanthi* (EU520248)	99	100
HS-Y-02			*Pestalotiopsis oxyanthi* (EU520248)	99	98
HS-J-04			*Pestalotiopsis oxyanthi* (EU520248)	99	99
HS-J-13	Diaporthales	*Diaporthe*	*Diaporthe phaseolorum* (GU066686)	99	100
HS-J-14			*Diaporthe phaseolorum* (GU066686)	99	100
HS-J-17		*Phomopsis*	*Phomopsis* sp. (KM229696)	99	99
HS-J-18			*Phomopsis* sp. (KM229696)	99	99
HS-J-02			*Phomopsis* sp. (KM229696)	100	100
HS-J-24			*Phomopsis* sp. (KM229696)	100	100
HS-J-25			*Phomopsis* sp. (GU066702)	99	100
HS-N-11		*Valsa*	*Valsa brevispora* (KJ780749)	99	99
HS-N-35			*Valsa brevispora* (KJ780749)	99	99
HS-G-02	Pleosporales	*Pyrenochaeta*	*Pyrenochaeta* sp. (JN253517)	97	99
HS-J-12		*Pleosporales*	*Pleosporales* sp. (GQ254684)	99	99
HS-Y-18		*Curvularia*	*Curvularia geniculate* (KT933687)	100	99
HS-Y-22		*Alternaria*	*Alternaria* sp. (KU663377)	100	98
HS-G-09	Capnodiales	*Cladosporium*	*Cladosporium* sp. (KJ958360)	100	99
HS-G-08			*Cladosporium halotolerans* (LN834369)	100	97

HS-N-07			*Cladosporium halotolerans* (KU059905)	100	100
HS-J-01	Botryosphaeriaceae	*Phyllosticta*	*Phyllosticta capitalensis* (KP998485)	100	100
HS-Y-07		*Lasiodiplodia*	*Lasiodiplodia theobromae* (KU291531)	99	96
HS-G-22			*Lasiodiplodia theobromae*(KT240140)		100
HS-Y-03	Glomerellales	*Colletotrichum*	*Colletotrichum* sp. (KT282716)	100	100
HS-Y-05			*Colletotrichum gloeosporioi*des (KC172072)	99	100
HS-Y-21			*Colletotrichum gloeosporioi*des (KC172072)	99	99

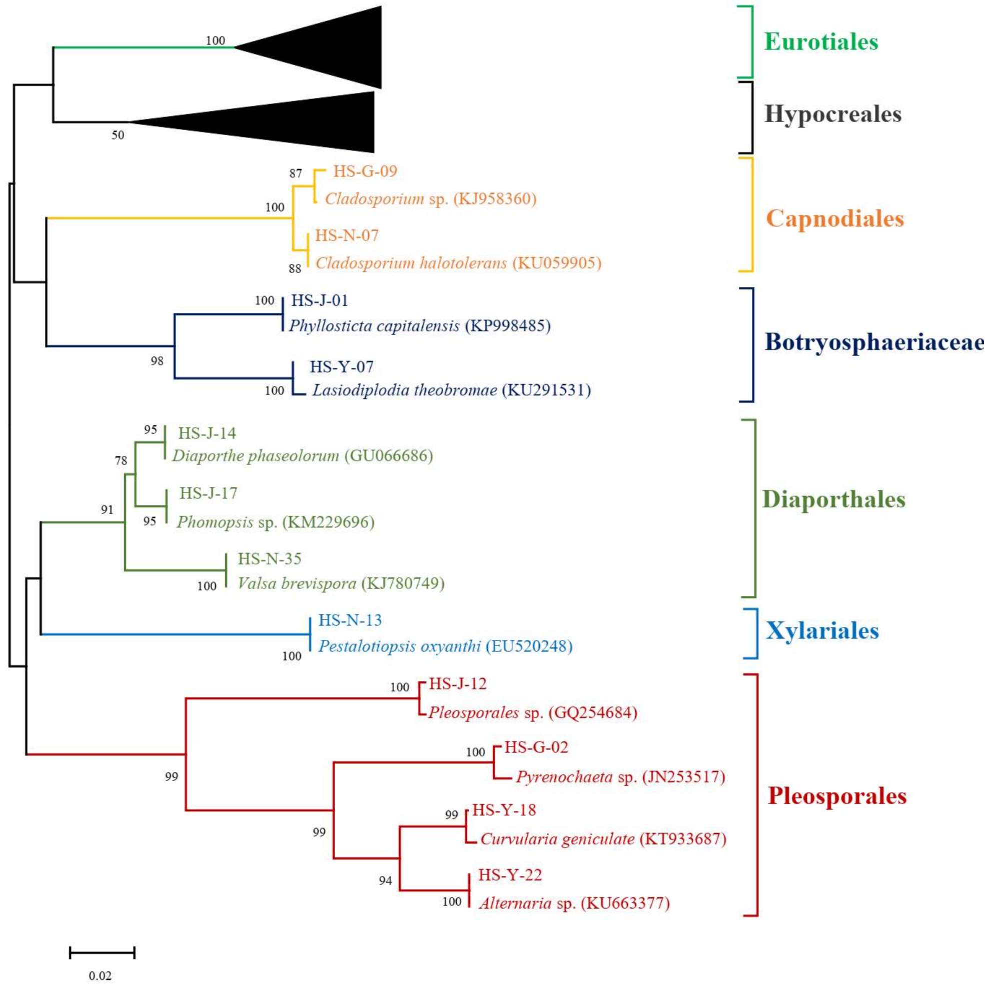

Figure S1. Neighbor-joining phylogenetic tree based on ITS sequences of cultured fungi associated with *Acanthus ilicifolius*. The values at each node represent the bootstrap values from 1,000 replicates, and the *scale bar*=0.02 substitutions per nucleotide.

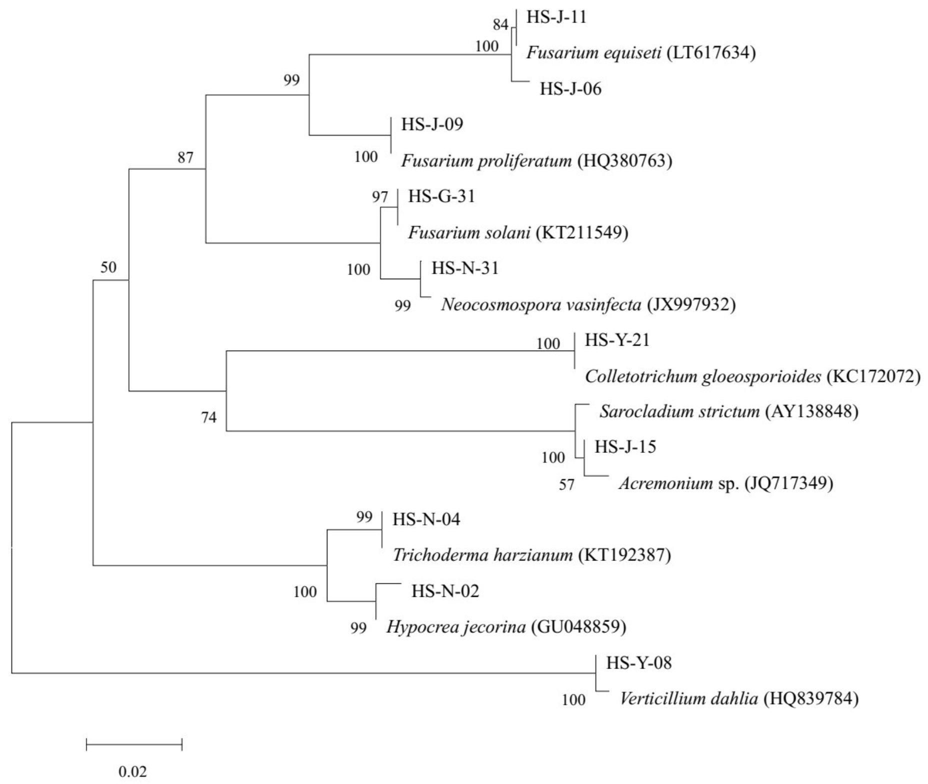

Figure S2. Phylogenetic tree of fungi in the order *Hypocreales* based on ITS gene sequence homology. The values at each node represent the bootstrap values from 1,000 replicates, and the *scale bar*=0.02 substitutions per nucleotide.

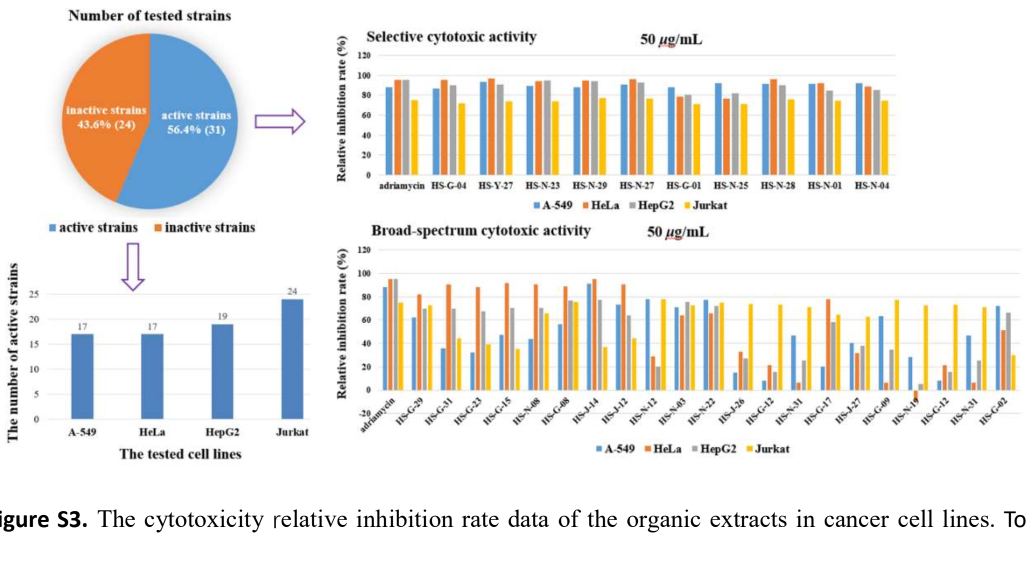

Figure S3. The cytotoxicity relative inhibition rate data of the organic extracts in cancer cell lines. To identify active strains for further research as potential cytotoxic strains, the relative inhibition rate of A-549, HeLa and HepG2 cell lines should be greater than 70 %, and the relative inhibition rate of Jurkat cell line should be greater than 60 %.

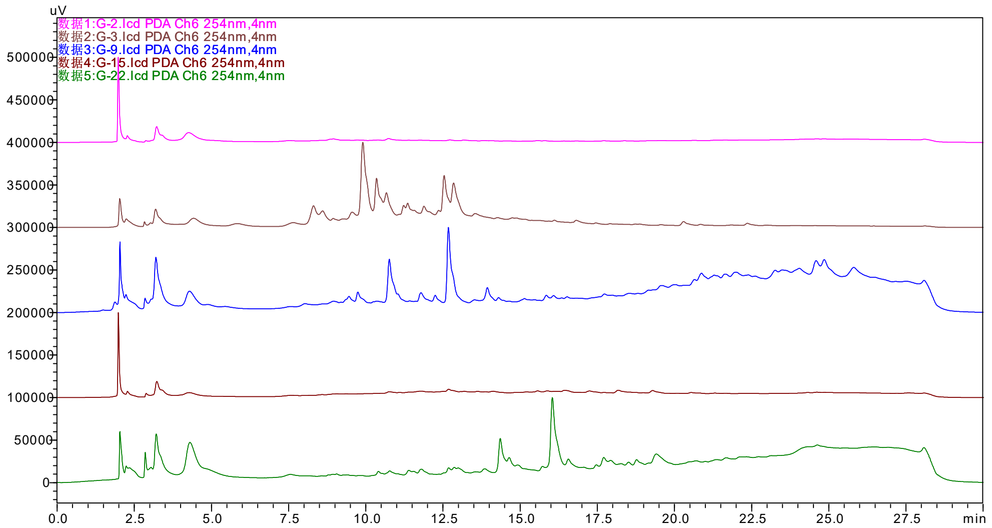
uV
数据1:G-2.lcd PDA Ch6 254nm,4nm
数据2:G-3.lcd PDA Ch6 254nm,4nm
数据3:G-9.lcd PDA Ch6 254nm,4nm
数据4:G-15.lcd PDA Ch6 254nm,4nm
数据5:G-22.lcd PDA Ch6 254nm,4nm
500000
450000
400000
350000
300000
250000
200000
150000
100000
50000
0
0.0 2.5 5.0 7.5 10.0 12.5 15.0 17.5 20.0 22.5 25.0 27.5 min

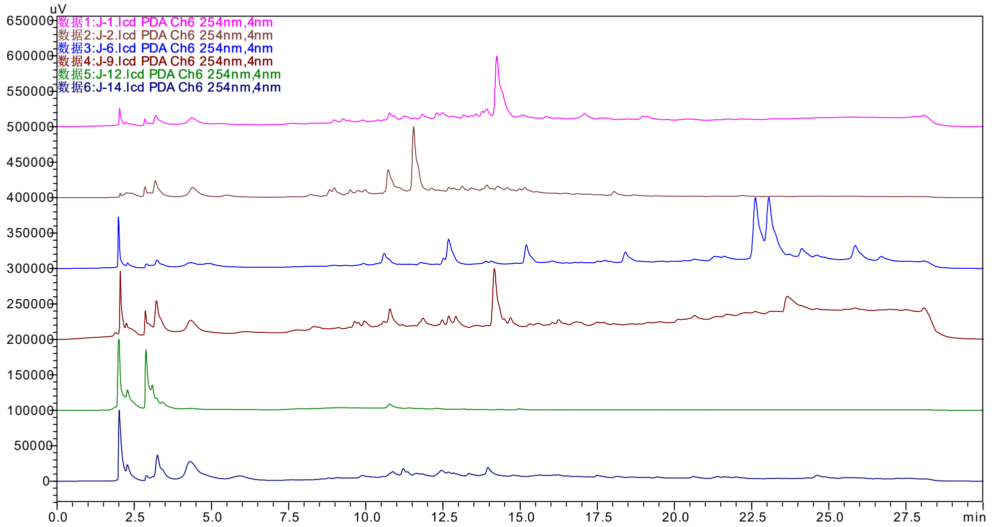
uV
数据1:J-1.lcd PDA Ch6 254nm,4nm
数据2:J-2.lcd PDA Ch6 254nm,4nm
数据3:J-6.lcd PDA Ch6 254nm,4nm
数据4:J-9.lcd PDA Ch6 254nm,4nm
数据5:J-12.lcd PDA Ch6 254nm,4nm
数据6:J-14.lcd PDA Ch6 254nm,4nm
650000
600000
550000
500000
450000
400000
350000
300000
250000
200000
150000
100000
50000
0
0.0 2.5 5.0 7.5 10.0 12.5 15.0 17.5 20.0 22.5 25.0 27.5 min

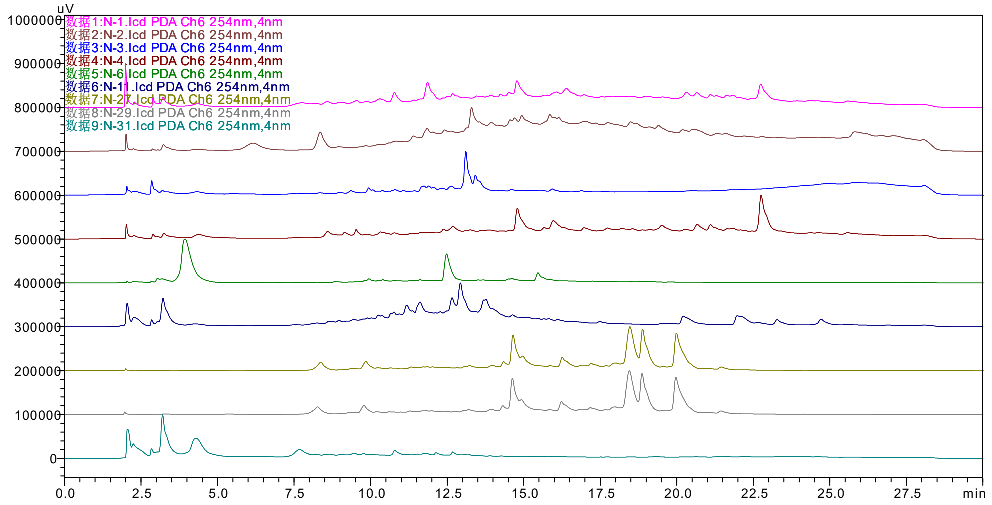
uV
数据1:N-1.lcd PDA Ch6 254nm,4nm
数据2:N-2.lcd PDA Ch6 254nm,4nm
数据3:N-3.lcd PDA Ch6 254nm,4nm
数据4:N-4.lcd PDA Ch6 254nm,4nm
数据5:N-6.lcd PDA Ch6 254nm,4nm
数据6:N-11.lcd PDA Ch6 254nm,4nm
数据7:N-27.lcd PDA Ch6 254nm,4nm
数据8:N-29.lcd PDA Ch6 254nm,4nm
数据9:N-31.lcd PDA Ch6 254nm,4nm
1000000
900000
800000
700000
600000
500000
400000
300000
200000
100000
0
0.0 2.5 5.0 7.5 10.0 12.5 15.0 17.5 20.0 22.5 25.0 27.5 min

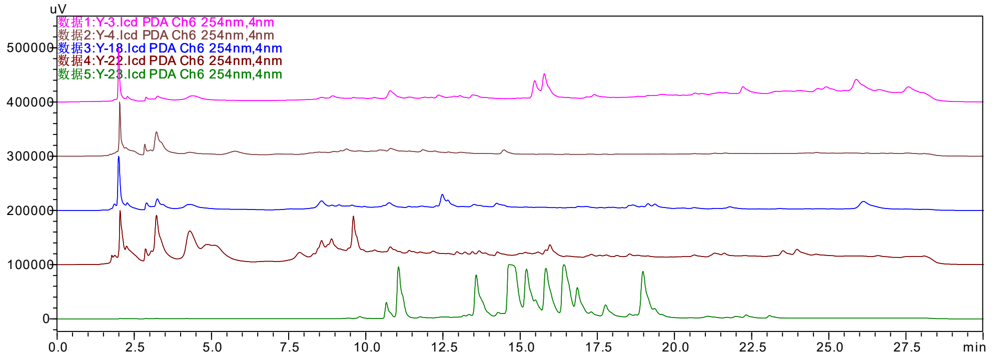

Figure S4. The fingerprint analysis of the organic extract of fungi from different species and genera. the organic extract of fungi (1 mg/mL, 50 μL) was analyzed by HPLC (Daojin LC-20AT, C_{18} column (250 mm × 5.0 mm) with a gradient program of MeCN−H_2O (0.1% formic acid) [0−1 min 10%, 1−20 min 5−100%, 20−23 min 100%, 23−25 min 5%, 25−30 min 5%; 1 mL/min]

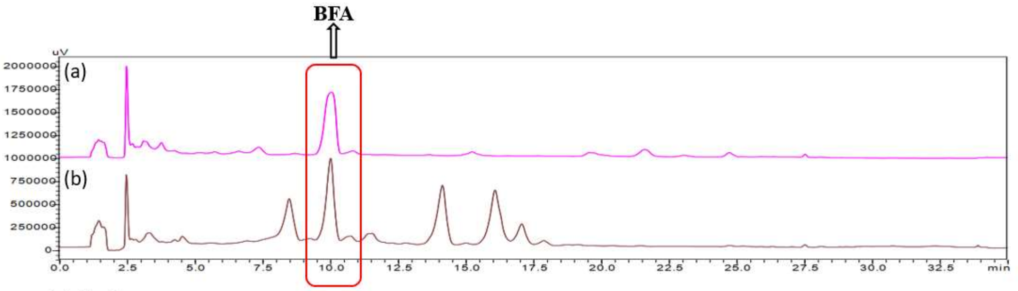

(a): Fr. 5
(b): the organic extract of *Penicillium* sp. (HS-N-27)

Figure S5. The fingerprint analysis of the organic extract of *Penicillium* sp. (HS-N-27).
